# A composite measure for patient-reported outcomes in orthopedic care: design principles and validity checks

**DOI:** 10.1007/s11136-023-03395-0

**Published:** 2023-03-24

**Authors:** Lukas Schöner, David Kuklinski, Alexander Geissler, Reinhard Busse, Christoph Pross

**Affiliations:** 1 Department of Health Care Management, Technical University Berlin, Straße des 17. Juni 135, 10623 Berlin, Germany; 2 Department of Health Care Management, University of St. Gallen, St. Gallen, Switzerland

**Keywords:** Patient reported outcome measures, Composite measure, Outcome quality measurement, Value-based health care, Composite measure development validation

## Abstract

**Background:**

The complex, multidimensional nature of healthcare quality makes provider and treatment decisions based on quality difficult. Patient-reported outcome (PRO) measures can enhance patient centricity and involvement. The proliferation of PRO measures, however, requires a simplification to improve comprehensibility. Composite measures can simplify complex data without sacrificing the underlying information.

**Objective and methods:**

We propose a five-step development approach to combine different PRO into one composite measure (PRO-CM): (i) theoretical framework and metric selection, (ii) initial data analysis, (iii) rescaling, (iv) weighting and aggregation, and (v) sensitivity and uncertainty analysis. We evaluate different rescaling, weighting, and aggregation methods by utilizing data of 3145 hip and 2605 knee replacement patients, to identify the most advantageous development approach for a PRO-CM that reflects quality variations from a patient perspective.

**Results:**

The comparison of different methods within steps (iii) and (iv) reveals the following methods as most advantageous: (iii) rescaling via z-score standardization and (iv) applying differential weights and additive aggregation. The resulting PRO-CM is most sensitive to variations in physical health. Changing weighting schemes impacts the PRO-CM most directly, while it proves more robust towards different rescaling and aggregation approaches.

**Conclusion:**

Combining multiple PRO provides a holistic picture of patients’ health improvement. The PRO-CM can enhance patient understanding and simplify reporting and monitoring of PRO. However, the development methodology of a PRO-CM needs to be justified and transparent to ensure that it is comprehensible and replicable. This is essential to address the well-known problems associated with composites, such as misinterpretation and lack of trust.

**Supplementary Information:**

The online version contains supplementary material available at 10.1007/s11136-023-03395-0.

## Introduction

The complex, multidimensional nature of healthcare quality makes quality measurement and transparency as well as provider and treatment decisions difficult for patients [1–5]. Patient participation in healthcare decision making presupposes that patients can understand quality information, which requires suitable quality measurement and reporting instruments [3, 6–9]. Patient-reported outcome measures (PROMs) are promising instruments that, in contrast to clinical indicators, measure patients’ own assessment of their current health status and enhance patient engagement [1, 4, 10–13]. PROMs are used to determine patient-reported outcomes (PRO), which are results of longitudinal comparison of individual PROM-scores, i.e., the change in individual PROM-scores attributable to a particular treatment. Despite their potential, the growing number of PROM makes it difficult to easily and comprehensively evaluate outcome quality [2, 12, 14–16]. Composite measures (CMs) can simplify complex, multidimensional data without sacrificing the underlying power of information [17–19].

A CM is a combination of two or more individual measures into one index, which captures multidimensional aspects that cannot be reflected by solely either of the individual measures [18]. In healthcare, CM provides a holistic picture of healthcare quality and can enhance ease of interpretation and comparability [20–22]. Next to benchmarking hospital or countries’ health system performance, CM can facilitate monitoring recovery paths and outcome quality as well as enhancing public accountability and quality transparency [23–26]. CM also plays an important role for the emerging value-based healthcare (VBHC) movement and allow researchers to better evaluate the results of clinical studies with several different PRO by a single outcome measure [27, 28]. Due to their advantages, healthcare CMs have already been widely applied in many different areas with different purposes [21, 22, 29–34]. However, there are also important downsides and challenges with CMs, which are controversially discussed in the literature [6, 17, 25]. Poorly constructed or opaque CMs can be particularly alarming as they have the potential to mask poor quality or deceive those who use them to make important policy and treatment decisions.

It is thus essential that the development methodology is clear and transparent to ensure that the CM is comprehensible and replicable. The chosen methodology is well justified and plausible and represents the relevant quality dimensions without losing or disguising important information [6, 35–37]. The development of CM, however, is often controversial, neither is there a gold-standard approach. Some guidelines for CM development are provided, e.g., by the OECD [35, 37] or, in a healthcare context, by Shwartz et al. [19]. However, so far CMs are mostly used to aggregate clinical outcomes. Furthermore, there is still a lack of studies that put these guidelines into practice.

In the present study, we combine the different considerations of OECD and Shwartz et al. to develop a patient-reported outcome CM (PRO-CM) applicable in routine orthopedic care and clinical studies. We propose a five-step development approach and highlight the need of transparency and justification of decisions in each step. We evaluate advantages and disadvantages of different rescaling, weighting, and aggregation methods, by utilizing PRO-data of primary hip and knee arthroplasty (PHA and PKA) patients. Due to the increasing case volume of hip and knee arthroplasty worldwide [38, 39] and since PROMs are already widely used in this field [40], the orthopedic setting provides a good example for illustrating development and benefits of a PRO-CM. Finally, we identify the most advantageous development approach for a multidimensional orthopedic PRO-CM that is transparent and replicable, combines all relevant sub-dimensions of PHA and PKA, and captures the relative differences and quality variations among these sub-dimensions. It is more sensitive to variations in the sub-dimensions that are most relevant for patients and partly compensates poorer outcomes in one dimension.

## Methods

### Data

We use data from the PROMoting Quality study [41], which provides PRO-data of 3,145 PHA- and 2,605 PKA patients of nine participating German hospitals between 2019 and 2021. Participants were adults undergoing an elective and primary hip or knee arthroplasty with pre-specified surgery codes (including total and partial arthroplasties) between 2019 and 2020. Exclusion criteria were emergency and life-threatening cases, ASA classification 4–6, and patients without direct or indirect access to an e-mail account or without a relative supporting the survey PROM response. The randomized-controlled trial was registered at the German Clinical Trials Register under trial number DRKS00019916 and examined the benefit of PROM-based patient follow-up based on the ICHOM standard set for Hip  and Knee Osteoarthritis with minor modifications [11, 42]: EQ-5D-5L captures Health-related Quality of Life (HRQoL) [43], Hip or Knee Osteoarthritis Outcome Score Physical Function Shortform (HOOS-PS or KOOS-PS) joint-associated problems and functionality [44], analogue pain scales assess pain in hip (left and right), knee (left and right), and lower back [42]. PROMIS Depression Shortform (PROMIS‐D‐SF) and Fatigue Shortform (PROMIS‐F‐SF) are included to capture mental health [45]. For a detailed description of the PROM, see Appendix I.

### Stepwise method for developing a composite measure

The study was preceded by a literature review on CM in general and in the healthcare context. The development approaches presented here are mainly based on current standards as provided by the OECD [35] and Shwartz et al. [19]. While we consider the OECD guidelines as a general toolkit for relevant technical and methodological issues (e.g., rescaling- and weighting- and aggregation-methods), the framework of Shwartz et al. provides relevant considerations in a healthcare context for creating hospital-level composites aggregating clinical outcomes. For the PRO-CM, we merge these considerations, adjust them to fit a patient-level orthopedic purpose and propose five PRO-CM development steps: (i) theoretical framework and metric selection, (ii) initial data analysis, (iii) rescaling, (iv) weighting and aggregation, and (v) sensitivity and uncertainty analysis [18, 19, 35]. Assessing risks and benefits of the different options we consider in step (iii) and (iv), we select a priori the most advantageous option with respect to the data structure and theoretical framework (i.e., “Model 1”) and compare the results to the other options (Model 2–5).

#### Theoretical framework and metric selection

The theoretical framework lays the foundation for a CM. It defines the quality construct (i.e., the phenomenon to be measured) and identifies its sub-dimensions [18, 35, 37]. Relevant quality indicators are identified so as to conform to the quality construct [46]. We select validated and well-established generic and disease-specific PROMs that align to the sub-dimensions of the quality construct.

#### Initial data analysis

We examine the PRO individually to analyze the underlying data structure (e.g., outliers and scale), which guides subsequent rescaling and weighting decisions. We plot descriptive statistics and compute Spearman’s rank correlations to check for collinearity [19, 24, 35]. Following similar studies [24, 37, 46], we consider indicators correlated higher than *r* = 0.7 to be merged into one variable to avoid redundancy or preponderance of one particular dimension [18, 36].

#### Rescaling

When indicators have different units of scale, rescaling on a common scale is required to allow comparison and aggregation. Different methods may produce different CM [19, 35] and it is not clear which method is favorable. Following Shwartz et al., we compare the two most widely used approaches for healthcare CM, i.e., z-score standardization and min–max normalization [19]. A priori we use z-score standardization (Model 1), as it preserves the relative differences, and extreme values and outliers don’t distort the mean but are recognized as exceptional performance. The z-score standardization transforms all individual measures on a dimensionless scale with mean = 0 and standard deviation (SD) = 1. Z-scores express how many SD an individual’s outcome is above or below the average of the population and is calculated as:

1 $$z=\frac{x-\mu }{\sigma }$$

where $x$ is the observed PRO of an individuum, $\upmu$ is the PRO-mean, and $\sigma$ is the SD. See Appendix II for an exemplary rescaling calculation.

#### Weighting and aggregation

Weights determine the contribution of each PRO to the CM [19, 35]. We consider three different weighting options: Equal weighting (EW), differential weighting (DW), and factor analysis (FA). Literature suggests that, without strong justification to use DW (e.g., not all sub-dimensions have the same importance in the quality construct), EW should be applied [19, 47]. EW assigns the same weight to all PRO, yielding a CM to which all PROs contribute equally. However, since orthopedic care primarily addresses joint functionality and HRQoL [48], we select a priori DW for Model 1, where physical dimensions and HRQoL receive higher weighting than mental dimensions. Ideally, DW perfectly reflects patient preferences which could be determined in a patient survey [19]. Since this exceeds the scope of this study, we approximate importance by each PROM-score’s improvement: The more a PROM-score has improved 12 months post-surgery, the higher its importance. The corresponding weights are determined by measuring the improvement of each sub-dimension in standard deviation units and calculating its proportion of the total sum of all improvements. Appendix III entails more detailed considerations of different weighting methods.

Aggregation combines the weighted individual PRO into the final PRO-CM. We consider a compensatory and a non-compensatory aggregation method. A priori we use additive aggregation (Model 1), a compensatory method where worse outcomes can be counterbalanced by better outcomes. Since both surgery and recovery process differ between PKA and PHA, two treatment-specific composites are generated. They are computed as:

2 $${\text{CM}}_{i}= \sum_{j=1}^{n}{w}_{j}{I}_{j}$$

where ${CM}_{i}$ is the CM for treatment $i$, ${w}_{j}$ is the weight of the *j*th rescaled PRO ${I}_{j}$.

#### Sensitivity and uncertainty analysis

In the sensitivity analysis, we calculate Pearson’s correlations between the resulting CM and the individual PRO to determine the PRO-CM’s sensitivity to quality variations among the sub-dimensions, i.e., the responsivity of the PRO-CM to changes in its sub-components. In the uncertainty analysis, we compare the results of models 1–5 to examine the impact of decisions in the chosen development approach and to analyze the associated uncertainties. For this, we convert the results of each model, in each of which we alter one decision, into patient rankings to illustrate the impact of altering a decision in the development process on the final result of a patient. The patient with the highest CM value gets assigned rank 1, the second highest rank 2, and so on. Patient rankings of our selected approach (Model 1) are compared to four alternative models (see Table 1). The greater the scatter between two compared models, i.e., the more the rankings of patients change depending on the model, the greater the impact of the corresponding changed development method. Models 2–5 are constructed as follows:

**Table 1:** Development approaches

CM	Rescaling	Weighting	Aggregation
Model 1	z-Score	DW	Additive
Model 2	Min–max	DW	Additive
Model 3	z-Score	EW	Additive
Model 4	z-Score	FA	Additive
Model 5	Min–max	DW	Geometric

*CM* Composite measure development approach*, z-Score* z-Score standardization*, min–max* min–max normalization*, DW* differential weighting, *EW* equal weighting, *FA* factor analysis

*Model 2* Rescaling PRO with min–max normalization method. Min–max normalization transforms the data’s original range to a common range from 0 to 1. It is calculated as:

3 $$m=\frac{x-{\text{min}}(x)}{{\text{max}}\left(x\right)-{\text{min}}(x)}$$

where $x$ is a PRO of an individuum, ${\text{min}}(x)$ is the minimum PRO, and ${\text{max}}(x)$ is the maximum PRO. Min–max normalization is more sensitive to outliers and can distort relative differences and mean values. However, due to a clearly defined boundary range, it has an intuitive appeal and strong interpretative power [19]. Also, when PROs are within a small interval, the range can be expanded to increase the effect on the CM [35].

*Model 3* Applying EW where all PROs contribute to the CM with the same importance. It is considered as the easiest strategy to implement, and it is not subject to any special interests and easily replicable by others [36, 49].

*Model 4* Using FA to derive weights statistically. The weight of each PRO is relative to the amount of variance in common with other PRO. An approach which is resistant to potentially intentional manipulation and often applied when a great amount of indicators exist [50–52].

*Model 5* Using geometric aggregation, a non-compensatory multiplicative approach that prevents poor outcomes from being compensated by good outcomes. It is computed as:

4 $${\text{CM}}_{i}= \prod_{j=1}^{n}{I}_{j}^{{w}_{j}}$$

where ${\text{CM}}_{i}$ is the CM for treatment $i$, ${w}_{j}$ is the weight of the jth rescaled PRO ${I}_{j}$ [35, 49, 53].

## Results

### Theoretical framework and metric selection

The PRO-CM is specific to PHA and PKA. It aims to reflect a multi-faceted picture of post-arthroplasty improvement in health as reported by patients, hence, does not entail clinical outcomes. Improvement in health (i.e., the PRO) is defined as PROM-score difference between hospital admission (HA) and the 12-month follow-up (12FU). To capture all patient-relevant aspects of post-arthroplasty improvement, we outline three main sub-dimensions of the PRO-CM. Those are general HRQoL (EQ-5D-5L) [43, 54], physical health (HOOS-PS, KOOS-PS, pain scales) [42, 44, 54], and mental health (PROMIS‐D‐SF, PROMIS‐F‐SF), as practical experience of healthcare experts and literature suggests that, although arthroplasty primarily addresses physical health, also mental health has a significant influence on patient recovery and is not sufficiently covered by EQ-5D-5L [41, 45, 48, 55, 56]. See Table 1 in Appendix I (Electronic Supplementary Material) for the PRO-CM dimensions and its sub-components.

### Initial data analysis

Table 2 shows summary statistics for hip and knee PROM-scores at HA and 12FU. EQ-5D-5L has mean of 0.62 (0.60) for PKA (PHA) patients at HA and 0.84 (0.87) at 12FU, with higher scores indicating better HRQoL. Scores range between -0.661 and 1, which covers the possible total range of EQ-5D-5L. All remaining PROM-scores have opposite directionality, with higher scores indicating worse outcomes. KOOS-PS (HOOS-PS) is at 43.3 (47.6) at HA and 26.0 (14.8) at 12FU with values between 0 and 100.

**Table 2:** Summary statistics of selected metrics for the PRO-CM

	PKA patients				PHA patients			
Number of cases	2605				3145			
Female, n (%)	1396 (53.6)				1781 (56.6)			
Age, mean (SD)	66.1 (9.1)				66.0 (10.4)			

PROM	HA		12FU		HA		12FU	
	Mean (SD)	Min (max)	Mean (SD)	Min (max)	Mean (SD)	Min (max)	Mean (SD)	Min (max)
HRQoL								
EQ-5D-5L	0.623 (0.253)	− 0.661 (1)	0.842 (0.201)	− 0.576 (1)	0.600 (0.257)	− 0.485 (1)	0.876 (0.173)	− 0.661 (1)
Physical health								
KOOS-PS	43.2 (12.9)	0 (100)	26.0 (14.2)	0 (91.8)	–	–	–	–
HOOS-PS	–	–	–	–	47.6 (16.3)	0 (100)	14.8 (14.6)	0 (100)
Pain-OJ	6.8 (2.0)	0 (10)	1.9 (2.0)	0 (10)	6.5 (2.1)	0 (10)	1.1 (1.7)	0 (10)
Pain-other	1.6 (1.4)	0 (9)	1.5 (1.4)	0 (8.5)	1.8 (1.5)	0 (9)	1.3 (1.4)	0 (10)
Mental health								
PROMIS- depression	49.4 (8.2)	41.0 (79.4)	47.7 (8.2)	41.0 (79.4)	49.8 (8.3)	41.0 (73.3)	47.3 (7.8)	41.0 (79.4)
PROMIS-fatigue	48.4 (9.8)	33.7 (75.8)	45.9 (9.4)	33.7 (75.8)	49.4 (9.9)	33.7 (75.8)	45.2 (9.1)	33.7 (75.8)

*PKA* Primary knee arthroplasty, *PHA* primary hip arthroplasty, *SD* standard deviation, *HA* hospital admission, *12FU* 12-month follow-up, *min* minimum value, *max* maximum value, *PROM* patient-reported outcome measure, *HRQoL* Health-related quality of life, *Pain-OJ* Pain in operated joint, *Pain-Other* Pain in non-operated joint

While Pain-OJ shows relatively high improvement for PKA (PHA) from 6.8 (6.5) at HA to 1.9 (1.1) at 12FU with a possible range from 0 to 10, Pain-Other is at a comparatively low level at HA and barely shows change during the recovery. Since neither PKA nor PHA appears to influence Pain-Other, this score is excluded. For mental health, PKA (PHA) patients have a mean level of depression of 49.4 (49.8) at HA and 47.7 (47.3) at 12FU with scores between 41 and 79.4, and a mean level of fatigue of 48.4 (49.4) at HA and 45.9 (45.2) at 12FU with values between 33.7 and 75.8.

Computing the PRO shows that physical health dimensions improved the most during recovery. On average, Pain-OJ was reduced by 1.55 (1.61) SD for PKA (PHA) patients, followed by an improvement in KOOS-PS (HOOS-PS) of 1.08 (1. 45) SD. HRQoL improved by 0.86 (1.04) SD for PKA (PHA) patients. Less variation is seen in mental health, with an average improvement in fatigue symptoms of 0.25 (0.43) SD and an improvement in depression symptoms of 0.21 (0.30) SD for PKA (PHA) patients [See Table 1 in Appendix III (Electronic Supplementary Material)]. Compared to PKA, PHA patients improve more during recovery in either dimension as they report worse PROM-scores at HA and better PROM-scores at 12FU. This is most evident in physical health, but also visible in HRQoL and mental health. Outliers exist for all PROM, with most extreme values of KOOS-PS (HOOS-PS). We found correlations between PROM albeit weak ones. EQ-5D-5L, which comprises mental health and pain sub-dimensions, is only weakly correlated (r ≤ 0.5) with mental health and pain. Since none of the correlations is > 0.7, each PROM has sufficient independent explanatory power to the purposes of this study.

### Rescaling

As a third step, we rescale via z-score standardization and compare it to min–max normalization (see Table 3). After z-score standardization, each PRO has mean = 0 and SD = 1. For equal directionality and an intuitive interpretation, each PRO is rescaled so that a higher value indicates more improvement. Values above 0 indicate more improvement than average in units of SD and vice versa. Upper and lower bounds can take (theoretically) infinite values, with values beyond ± 3 usually considered to be outliers.

**Table 3:** Rescaling of patient-reported outcomes (PRO)

	PKA				PHA			
	z-score standardization		Min–max normalization		z-score standardization		Min–max normalization	
	Mean (SD)	Min (max)	Mean (SD)	Min (max)	Mean (SD)	Min (max)	Mean (SD)	Min (max)
Δ EQ5D	0 (1)	− 4.9 (3.7)	0.6 (0.1)	0 (1)	0 (1)	− 5.5 (4.1)	0.6 (0.1)	0 (1)
Δ KOOS	0 (1)	− 4.8 (5.6)	0.5 (0.1)	0 (1)	–	–	–	–
Δ HOOS	–	–	–	–	0 (1)	− 4.0 (3.6)	0.5 (0.1)	0 (1)
Δ Pain	0 (1)	− 4.6 (1.9)	0.7 (0.2)	0 (1)	0 (1)	− 4.4 (1.8)	0.7 (0.2)	0 (1)
Δ Dep	0 (1)	− 5.0 (3.5)	0.6 (0.1)	0 (1)	0 (1)	− 5.0 (3.4)	0.6 (0.1)	0 (1)
Δ Fat	0 (1)	− 4.0 (4.2)	0.5 (0.1)	0 (1)	0 (1)	− 4.8 (4.0)	0.6 (0.1)	0 (1)

*PKA* Primary knee arthroplasty, *PHA* primary hip arthroplasty, Δ patient-reported outcomes (i.e., PROM-Score Changes between Hospital Admission and 12-month Follow-up), *SD* standard deviation, *min* minimum value, *max* maximum value, *Pain* Pain in operated joint, *Dep* PROMIS depression, *Fat* PROMIS fatigue

Min–max normalization transforms all PRO onto the same scale from 0 to 1 (Model 2). Since especially negative outliers are present, most normalized PROs have mean values greater than 0.5, indicating how min–max normalization is affected by outliers. Caution must be exceeded in interpretation as the worst PRO defines the lower boundary and a normalized value of 0 can indicate PRO-deterioration.

### Weighting and aggregation

**Fig. 1:**
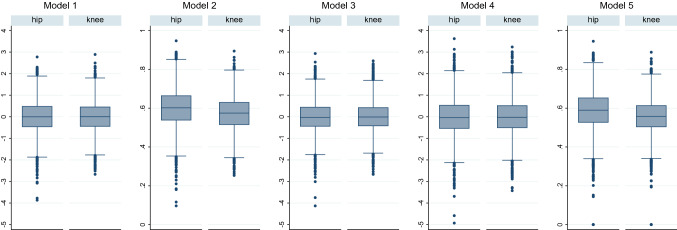
Distribution of the PRO-CM Models. Figure 1 shows the PRO-CM (Model 1) and the four alternative development models: Model 1: z-score standardization, differential weights, additive aggregation; Model 2: min–max normalization, differential weights, additive aggregation; Model 3: z-score standardization, equal weights, additive aggregation; Model 4: z-score standardization, factor analysis, additive aggregation; Model 5: min–max normalization, differential weights, geometric aggregation. The left box of each panel shows results for PHA, the right side for PKA patients. Z-Score standardized CM have a scale from − 5 to 4 while minmax normalized CM have a scale from 0 to 1

The initial data analysis shows physical health dimensions to improve the most, followed by HRQoL and mental health dimensions. Consequently, for Model 1, estimated weights are 0.3 for each physical health sub-dimension, 0.2 for HRQoL, and 0.1 for each mental health sub-dimension [for a more detailed description, see Table 1 in Appendix III (Electronic Supplementary Material)]. This is in line with our assumption that physical health should be assigned more importance than mental health. Contrarily, EW assigns the same weight to each PRO, i.e., 0.2 (Model 3), while FA (Model 4) derives the weights statistically and assigns more weight to mental health. Figure 1 shows the boxplots of the five resulting PRO-CM models after aggregation of the weighted indicators.

The PRO-CM in Model 1 has a mean of 0 and SD of 0.73 for both PHA and PKA patients. Like the z-scores, it can take theoretically infinite values. Patients take values between ± 2 while PHA patients show more negative outliers with less than -3. Model 2 yields a CM with mean of 0.57 (0.60), SD of 0.09 (0.1), and a range from 0.25 to 0.89 (0.1 to 0.95) for PKA (PHA) patients. Model 3 shows a similar mean and SD as in Model 1, however, slightly contracts the range for PKA patients while expanding the range for PHA patients. Model 4 in general yields a higher SD and larger range and more extreme outliers for PKA and PHA patients with both having a mean of 0. Lastly, Model 5 has mean of 0.56 (0.59) and SD 0.09 (0.1) for PKA (PHA) patients with minimum values of 0, where at least one PRO was equal to 0.

### Sensitivity and uncertainty analysis

The sensitivity analysis shows that, although in Model 1 the weights for pain-OJ and KOOS-PS (HOOS-PS) are equal, there are minimal differences in the sensitivity of the PRO-CM to variation in these PRO. Correlations (see Table 4) show the highest sensitivity in PKA (PHA) to changes in physical functionality measured by KOOS-PS (HOOS-PS) with *r* = 0.81 (*r* = 0.82). Thus, a change in KOOS-PS (HOOS-PS) contributes most to a change in the PRO-CM compared to other PRO. In contrast, Pain-OJ is weakly correlated with PRO-CM and has a similar level of correlation as HRQoL assessed by EQ-5D-5L. The least sensitivity is shown to change in both mental health dimensions with correlations around *r* = 0.5.

**Table 4:** Sensitivity of the PRO-CM and alternatives to patient-reported outcomes (PRO)

	PKA (PHA)					
	Δ EQ-5D-5L	Δ KOOS-PS	Δ HOOS-PS	Δ Pain-OJ	Δ PROMIS-D	Δ PROMIS-F
Model 1	0.75 (0.77)	0.81	0.82	0.74 (0.77)	0.54 (0.50)	0.55 (0.54)
Model 2	0.73 (0.73)	0.76	0.81	0.81 (0.82)	0.52 (0.47)	0.53 (0.51)
Model 3	0.76 (0.77)	0.74	0.74	0.62 (0.65)	0.69 (0.67)	0.70 (0.69)
Model 4	0.78 (0.81)	0.76	0.77	0.56 (0.62)	0.70 (0.63)	0.71 (0.68)
Model 5	0.73 (0.73)	0.79	0.84	0.73 (0.77)	0.54 (0.49)	0.57 (0.53)

*PKA* primary knee arthroplasty, *PHA* primary hip arthroplasty, Δ patient-reported outcomes, i.e., PROM-Score changes between hospital admission and 12-month follow-up

This is similar in Model 2. However, the min–max normalization leads to pain-OJ becoming the largest contributor for changes in the PRO-CM, whereas it becomes somewhat less sensitive to KOOS-PS (but remains stable for HOOS-PS). Yet, this CM remains most sensitive to changes in physical health dimensions, followed by changes in HRQoL and finally in mental health dimensions. The correlations are more balanced in Model 3, with slightly higher sensitivity to changes in KOOS-PS (HOOS-PS) and HRQoL than to changes in pain-OJ and mental health dimensions. Model 4 results in a CM that is most sensitive to changes in HRQoL and KOOS-PS (HOOS-PS). Mental health dimensions gain importance, while pain-OJ has the weakest correlation. Lastly, Model 5 shows very similar results to the additive approach in Model 1.

**Fig. 2:**
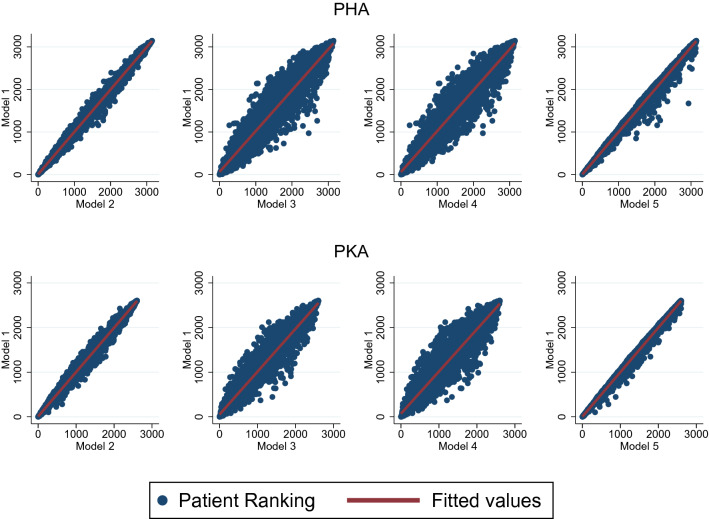
Relation between Model 1 and alternative Models. Figure 2 shows the relation between the PRO-CM (Model 1) and the four alternative development models: Model 1: z-score standardization, differential weights, additive aggregation; Model 2: min–max normalization, differential weights, additive aggregation; Model 3: z-score standardization, equal weights, additive aggregation; Model 4: z-score standardization, factor analysis, additive aggregation; Model 5: min–max normalization, differential weights, geometric aggregation. The results of the respective development models are converted into patient rankings to show the impact of altering a decision in the development process can have on the final result of a patient in comparison to other patients. The closer the scattering of patient rankings is to the fitted line, the more strongly the two models compared are correlated and accordingly smaller is the impact of their different calculation method on the final result

Results of the uncertainty analysis are illustrated in Fig. 2, which shows the relation between the PRO-CM in Model 1 and the four alternative models. The y-axis represents Model 1 patient rankings and the x-axis patient rankings of the respective alternative approach. Correlations between Model 1 and the alternative approaches are generally high, with values between *r* = 0.95 and *r* = 0.99. In particular, there are only minor changes in patient ranking between z-score standardization and min–max normalization (*r* = 0.99), when the same weighting scheme is applied (Model 1 vs. Model 2). Altering the rescaling method does not lead to any significant distortions in our case. Also altering between additive and geometric aggregation has no significant effect on the resulting PRO-CM (Model 1 vs. Model 5). The biggest discrepancies arise when applying different weighting schemes, i.e., EW (Model 1 vs. Model 3; *r* = 0.96) and FA (Model 1 vs. Model 4; *r* = 0.95). Hence, while aggregation and rescaling approaches play a negligible role for the PRO-CM, it is most sensitive to the weighting methods.

## Discussion

In this study, we have proposed a development approach of a patient-centered PRO-CM for PKA and PHA patients and compared it to four alternative models. The PRO-CM is robust towards different aggregation and rescaling methods, while applying different weighting schemes can have a greater impact on the final result. We consider the approach with z-scores, DW, and additive aggregation as most advantageous with respect to the data properties and the theoretical framework (Model 1). Z-scores do not distort the mean by preserving the relative differences and extreme values are acknowledged as exceptional performance, while min–max normalization (Model 2) is heavily affected by outliers [35]. DW assigns more importance to physical health dimensions that play an important role in PKA and PHA recovery [48]. EW (Model 3) should be applied when there is no strong justification to apply DW, while FA (Model 4) is rather suitable when a great number of different indicators are combined to one score [50, 52]. Additive aggregation allows, to some extent, poor outcomes to be compensated by good outcomes. In some cases, depressive symptoms were already at a low level and thus an improvement of 0 took place. With non-compensatory aggregation (Model 5), this would lead to a final CM value of 0 despite a very large improvement in physical dimensions.

As shown in the sensitivity analysis, the PRO-CM is capable of measuring relevant quality variations among sub-dimensions. The information from the individual PRO is still contained, but for outcome comparisons, only one metric must be considered instead of many different metrics. The PRO-CM can therefore empower patients, as it simplifies the monitoring of their recovery and enables them to make meaningful provider and treatment choices through enhanced comprehensibility [21, 23]. Physicians can track their patients’ recovery and quickly respond to health deteriorations with treatment adjustments [25, 26]. It is also eligible for public reporting, since assessing and ranking provider performance is facilitated [2, 3]. Reducing the outcome-side of any cost–benefit consideration to one-multidimensional metric also might aid health policy decisions, whether to calculate and present the cost-effectiveness of new forms of treatments, or to determine patient-value in the emerging VBHC considerations [27, 28, 58].

As with any CM, there are some specific and some more general limitations [6]. First, since z-scores have no clear boundaries, interpretation of z-score-based CM is difficult and not intuitive. Interpretability and comprehensibility can be enhanced by transforming the PRO-CM, e.g., to a scale from 0 to 100 (T-score transformation). Other possible approaches, such as ranking or 5-star classification, have been excluded in advance, as these methods entail a loss of information [19, 35]. However, intuitive visualization formats are highly relevant for the presentation of health data, such as the PRO-CM, and need to be discussed in a separate study [57]. Next, ideally DW perfectly reflects the preferences of patients [19]. Approximating preferences from PRO is a strong assumption and is certainly not the same for all patients. However, without knowing the true preferences, it is difficult to evaluate otherwise. Further, in this study, a complete dataset without missing values from a clinical study was used. However, in most datasets, missing values are present for which appropriate imputation methods must be applied to avoid selection bias [35]. Lastly, we illustrated the benefits of a PRO-CM with available data from the PROMoting Quality study. For broad application and realizing full potential, cross-clinic PRO-data must be available nationwide. This underlines the urgency of advancing broader PRO-measurement and usage along the patient pathway, which, at least in Germany, is still in its infancy [5]. As is, the PRO-CM developed here will primarily be applied in the evaluation of clinical trials.

Generally, opaque construction methods or individual components of poor quality can cause misinterpretation and, hence, mislead patients or trigger overly simplistic treatment, management. or policy decisions [19, 24]. When the construction methodology and its robustness are not transparently displayed, CM can easily and intentionally be skewed [6]. They can be misused for individual goals and purposes if intentionally formed for specific desired policies. It can lead to disguising very poor performance in one dimension by better performance in another and, hence, complicates the task of making targeted interventions to improve individual dimensions [6, 17]. Since a specific weighting of the underlying indicators is applied, conflicts might appear with different preferences of patients and admitting physicians [3, 59]. Although the threats and problems are widely known, CMs are often presented without going into more detail about the development process [6]. In this study, we addressed these problems and enable replicability by justifying each step in the development.

## Conclusion

We provide a transparent, stepwise development approach for a multidimensional PRO-CM that can effectively capture quality variations in orthopedic surgery. Combining multiple PRO provides a simplified but holistic picture of patients’ health status while single PRO only provides information about a specific dimension. By reducing information overload, using a PRO-CM can enhance the benefits of quality transparency. However, to avoid misleading of policy, treatment, or provider decisions, the development methodology of a PRO-CM, as presented here, needs to be justified and transparent to ensure that the composite is comprehensible and replicable. Only in this way can the known problems of CM be counteracted and their full potential unfolded, which should serve one thing above all else, the promotion of quality in healthcare.

## Supplementary Information

Below is the link to the electronic supplementary material.

Supplementary file1 (DOCX 90 KB)
