# Supplementary material for: A composite measure for patient-reported outcomes in orthopedic care: design principles and validity checks
Source: Qual Life Res. 2023 Mar 24;32(8):2341–51. doi: 10.1007/s11136-023-03395-0 (PMC10329084; doi:10.1007/s11136-023-03395-0)
Supplement: Supplementary file 1 — Supplementary file1 (DOCX 90 KB) [file 11136_2023_3395_MOESM1_ESM.docx]

# Appendix I

*EQ-5D-5L*

EQ-5D-5L is a generic PROM that captures HRQoL in five dimensions, i.e. Mobility, Self-Care, Usual Activities, Pain/Discomfort, and Anxiety/Depression [1]. Patients rate each dimension by five levels of functioning. This yields 55 = 3125 unique health states, all individually indexed on the country-specific preferences of the average population for many countries around the world. In context of this article, the German value set is used. Typically, the EQ-5D-5L values range from -0.661 to 1, where the preference-based scores indicate states worse than death (<0) to 1 (full health), anchoring death at 0. A higher score indicates better HRQoL. Designed for self-completion by respondents and applicable for many purposes, it is mainly used for clinical trials, population health surveys and routine outcome measurement [1–4].

*HOOS-PS and KOOS-PS*

The Hip and Knee injury and Osteoarthritis Outcome Score Physical Function Shortform (HOOS-PS and KOOS-PS) assess hip or knee and associated problems and functionality evaluating both, short-term and long-term consequences of knee and hip injuries. They consist of five sub-domains: Pain, other Symptoms, Function in daily living, Function in sport and recreation and hip and knee-related quality of life. The items are coded on a likert-scale from 0 (no problems) to 4 (severe problems) and subsequently converted to a scale from 0 to 100 with higher scores indicating worse symptoms and, vice versa, lower scores indicating better functioning [5].

*PROMIS Fatigue and Depression*

Even though the EQ-5D-5L already in part captures depression and anxiety dimensions, also the generic PROMIS Fatigue and Depression Short Forms (PROMIS F-SF and PROMIS D-SF) are included to assess the patients’ mental health. PROMIS‐F‐SF and PROMIS‐D‐SF (version 4a) are 4‐item questionnaires developed for use with the general population and individuals living with chronic conditions [6]. Patients assess their fatigue and depression within the prior seven days by rating items on a 5‐point scale. The raw scores are converted into t‐scores on a scale with hypothetical values from 0 (no fatigue/depression) to 100 (severe fatigue/depression) [7]. The rationale behind including PROMIS‐F‐SF and PROMIS‐D‐SF is based on the practical experience of experts and the literature, which suggests that these dimensions have a significant influence on patient recovery and are not sufficiently covered by the EQ-5D-5L [8].

*Pain*

In addition to the pain dimensions captured by the EQ-5D-5L, HOOS, and KOOS, analogue pain scales from the ICHOM standard set were used to assess patients' joint-specific pain in the knee, hip, and lower back, providing more detailed insights into the resulting problems. Patients can rate their hip, knee, and back-related pain on an analogue scale of 0-10. The individual items are combined into an overall pain score where 0 corresponds to no pain and 10 to very severe pain [9].

Table 1

| **PRO-CM Dimension** | **PROM** | **PROM Sub-Dimensions** | **Sub-Dimension Levels** |
| --- | --- | --- | --- |
| HRQoL | EQ-5D-5L | Mobility | 5 |
|  |  | Self-Care | 5 |
|  |  | Usual Activities | 5 |
|  |  | Pain/Discomfort | 5 |
|  |  | Anxiety/Depression | 5 |
| Physical Health | KOOS-PS | Rising from bed | 5 |
|  |  | Putting on socks/stockings | 5 |
|  |  | Rising from sitting | 5 |
|  |  | Bending to the floor | 5 |
|  |  | Twisting/pivoting on injured knee | 5 |
|  |  | Kneeling | 5 |
|  |  | Squatting | 5 |
|  | HOOS-PS | Sitting | 5 |
|  |  | Descending stairs | 5 |
|  |  | Getting in/out of bath or shower | 5 |
|  |  | Twisting/pivoting on loaded leg | 5 |
|  |  | Running | 5 |
|  | Pain | Hip Left | 10 |
|  |  | Hip Right | 10 |
|  |  | Knee Left | 10 |
|  |  | Knee Right | 10 |
|  |  | Lower Back | 10 |
| Mental Health | PROMIS-D-SF | Felt worthless in the last 7 days | 5 |
|  |  | Felt helpless in the last 7 days | 5 |
|  |  | Felt down in the last 7 days | 5 |
|  |  | Felt hopeless in the last 7 days | 5 |
|  | PROMIS-F-SF | Felt fatigued in the last 7 days | 5 |
|  |  | Starting something is difficult because of fatigue | 5 |
|  |  | General extent of exhaustion in the past 7 days | 5 |
|  |  | General extent of fatigue in the past 7 days | 5 |

# Appendix II

In the following, an overview of the calculation of either rescaling method based on theoretical examples is provided.

*Z-Score Standardization*

One of the most common approaches of related studies to make differently scaled metrics comparable is converting them into z-scores. Z-score standardization transforms all individual indicators on a common scale with mean of zero and standard deviation of one. The z-scores are calculated for each patient and indicate how many standard deviations an observation is below or above the mean. It is calculated with the following formula:

$$z_{i}=\frac{x_{i}-\mu_{Prom}}{\sigma_{Prom}}$$

where $\mathbf{x}_{\mathbf{i}}$ is the value of one indicator of individuum ***i*** and $\boldsymbol{\mu}_{\boldsymbol{Prom}}$ and $\boldsymbol{\sigma}_{\boldsymbol{Prom}}$ are the mean and standard deviation of the corresponding indicator. Z-scores reflect the distance of the data according to the mean value of a reference population in units of standard deviation. Thereby, it is important to define the reference population [10]. Depending on the desired level of comparison, the mean and standard deviation can be calculated on basis of different reference populations. For example, on patient level, outcomes of patients of one facility are compared. Accordingly, mean and standard deviation are computed on basis of all patients of this facility. On hospital level (i.e. comparing the performance across facilities) mean and standard deviation are calculated for all patients of all facilities that are to be compared. Country level comparisons (i.e. comparing performance across countries) take place on basis of mean and standard deviation of all patients of all countries of interest.

To illustrate the calculation and interpretation with a simple example, assumed that (hip) patient ***i*** has an EQ-5D-5L outcome of 0.51 with the facility mean of 0.745, then the corresponding z-score is:

$$z_{i}=\frac{0.51-0.745}{0.235}=-1$$

which indicates that patient i’s EQ-5D-5L outcome at time *t* is one standard deviation below the population’s mean. Now assumed that the same patient’s standardized HOOS z-score is -1 as well (keeping in mind the different directionality, i.e. a lower HOOS Score indicates better functionality) one can say, even though patient i’s health related quality of life is 1 SD below average, the functionality of the new hip is 1 SD better than average, a statement that is not readily possible when comparing the raw data 0.51 vs. 17.1.

Finally, in case of different directionalities as given in the example, it is important to invert the scores so that all z-scores above 0 indicate better than average values and all z-scores below 0 worse than average values. This is simply done by multiplying the standardized indicator to be changed by -1.

*Min-Max Normalization*

Rescaling indicators applying the z-score approach doesn’t define a specific range. Depending on how extreme the values of the indicator are, the z-scores can take on indefinite values in both directions. Sometimes, however, especially to improve interpretability, it is desired to transform the components to a specific scale, e.g. a scale from 0 to 100. This is, inter alia, done by min-max normalization.The min-max normalization uses proportions of the range of the data and transforms the original indicators on an identical scale from 0 to 1. Normalized indicators are calculated for each individuum by subtracting the minimum value from the observed value and dividing by the range of the data (i.e. maximum value – minimum value):

$$I_{i}=\frac{x_{i}-{min}_{Prom}}{{max}_{Prom}-{min}_{Prom}}$$

Looking back on the example of before, the patient’s raw data is transformed on the scale from 0 to 1 with values 0.7 for the EQ-5D-5L and 0.171 for the HOOS-PS. In this case the inverted directionality of the HOOS-PS is changed by 1 - 0.171 = 0.829 or by exchanging the minimum with the maximum value in the formula. Thus, the patient’s health related quality of life is at 0.7 while the functional status of the new hip is at 0.829 of a possible maximum of 1.

# Appendix III

Identifying appropriate weights is essential as weights determine the relative importance with which each indicator contributes to the composite by modifying their original value. Further, weights also symbolize the trade-off ratios between the different indicators, provided they are conceived as substitutable [11]. This makes this step prone to manipulation and misleading, e.g. for policy-makers by altering or lowering weights to improve overall performance. In practice, justification and transparency of weighting schemes often is not provided sufficiently [12]. The selected weighting scheme, however, should not only meet the purpose of the quality construct but also needs to be transparent and justified [11, 13].

Existing literature provides a variety of different weighting approaches, such as participatory approaches that incorporate various stakeholders (e.g. experts, patients, politicians) or statistical approaches, such as Principal Component (PCA) and Factor Analyses (FA). Each approach is associated with differing pros and cons and their application depends on the particular issue. Without strong justification to use differential weights, equal weights should be applied [14, 15]. Equal weighting implies that each indicator is considered to be equally important to measure health care quality, hence, the effect of each indicator is the same on the resulting composite measure. It is the easiest and most straightforward strategy to implement, is not subject to any vested interests and can very well be replicated by others [11, 16].

Even though the use of equal weights is simple and intuitive, and isn’t based on further assumptions, we have identified two problems in our context that could speak against its use. Firstly, since the combined indicators are partly composites themselves, applying equal weighs could lead to double weighting. This is especially relevant for the mental health dimensions captured by the EQ-5D-5L and the PROMIS Depression and Fatigue Scores. EQ-5D-5L combines, among others, depression- and anxiety-aspects in one index. Since it has been shown that the recovery process of patients is also influenced by mental health, but this dimension is not sufficiently covered by the EQ5D, PROMIS depression and fatigue scores were added. Equal weighting could now lead to predominating of mental health dimensions in the composite. Secondly, and associated with the first point, discussions within a team of experts (consisting of health scientists and physicians) raised doubts as to whether it is appropriate in this patient-centered, orthopedic setting to assign the same weight to each of the captured health-dimension. In particular, it was questioned if mental health such as depression and fatigue should be weighted as much as physical function when the orthopedic composite aims to reflect the interests of the hip and knee patients. After joint replacements, functional aspects are expected to play a more important role than mental dimensions. Accordingly, it would be appropriate to assign higher weighting to the function-specific HOOS and KOOS. Since the resulting composite aims to reflect value-based healthcare and, with this, should represent health care quality from the patients’ point of view, it is necessary to identify the indicators what matter most to patients having undergone a hip or knee replacement. In such cases, it is common to take differential weights, with greater weighting on the relevant dimensions (see e.g. [17, 18]). Differential weights are often determined in an multistage budget allocation process, similar to Delphi [17, 19]. Ideally, patients would assign points to the individual indicators according to their preferences in a budget allocation. The weightings are then calculated according to the distribution of points. However, since a patient survey would exceed scope of this study, patient preferences were assessed as relative improvements of the indicators between hospital admission and 12 months follow ups, hypothesizing the more time-sensitive an indicator is, the more it matters to the patient. With this, indicators with more variation will be more emphasized in the composite measure compared to less variable indicators. Corresponding weights are derived from these relative improvements, yielding a composite where physical function dimensions are assigned with greater weights then mental health dimensions. In this approach we see two advantages. First, the weights are derived from the data-structure and, hence, are resistant towards manipulation and opinions of different stakeholders. Second, results meet the expectations that in this setting physical dimensions matter to patients in a higher degree then mental health dimensions.

Factor Analysis (PCA)

Some studies [17, 20–23] use factor analysis as a statistical approach to derive the weights for a composite measure. FA identifies the common variance amongst a set of observed indicators, and creates a factor (i.e. index) comprised of that common variance. The factor scores are calculated with a linear equation that incorporates a weighted contribution of each of the variables that are included in the analysis. The contribution (i.e. weight) of each variable is relative to the amount of variance in common with the other variables. Factor analysis is used for theory development, psychometric instrument development, and data reduction [24]. It works by reducing the number of variables while maximizing the proportion of variance covered.

The theory behind factor analysis is that two (or more) indicators are correlated because of an underlying "factor" causing the performance on the two indicators to be related. The factor cannot be measured directly, but is only seen in the indicators that can be measured. If the results of the analysis indicate that there is one underlying factor, then a single factor score could be used to describe the entire set of indicators. Because this factor influences all rates, a region's rate for one indicator can tell you something about their rates on the other related indicators [25].

The main point is that factor analytic theory is about accounting for the covariation between observed variables. When observed variables are correlated with each other, factor analytic theory says that the correlation is due, at least in part, to the influence of common latent variables [24].

The limitations of factor analysis can be listed as follows: the type of symptom measures used (i.e. their breadth or narrowness of scope); the type of factor analysis and factor solution chosen; the nature of the sample selected (i.e. the narrowness or breadth of people with specific psychotic disorders examined); the phase of illness (chronic or acute), and the time frame measured by a scale (see, amongst others, Peralta & Cuesta, 2001; Blanchard & Cohen, 2006). Moreover, cross-sectional factor analytic studies assume stability of signs and symptoms (see, e.g. Shevlin et al., 2017). Finally, patients with affective psychosis, psychotic disorder due to another medical condition or substance misuse (intoxication and withdrawal) have typically been omitted from factor analytic studies—though these are all conditions where psychotic phenomena can occur. The minimum sample size needed for factor analysis is a source of confusion, with common recommendations having little empirical support. Minimum sample size depends on the size of communalities (i.e. variance in indicator variables explained by the factors, which should be large) and the number of variables per factor (the more variables per factor the better) [26].

*Table 1: Improvements of PROM-Scores in SD and results of differential weighting*

|  | Knee | | | Hip | | |
| --- | --- | --- | --- | --- | --- | --- |
|  | Δ SD | Ind. Weight**^1^** | Dim. Wight**^2^** | Δ SD | Ind. Weight**^1^** | Dim. Wight**^2^** |
| EQ-5D-5L | 0.9 | 0.23 | 0.20 | 1.0 | 0.22 | 0.20 |
| KOOS-PS | 1.1 | 0.28 | 0.30 | - | - | 0.30 |
| HOOS-PS | - | - | - | 1.5 | 0.30 | - |
| Pain-OJ | 1.5 | 0.38 | 0.30 | 1.6 | 0.33 | 0.30 |
| PROMIS-D | 0.2 | 0.05 | 0.10 | 0.3 | 0.06 | 0.10 |
| PROMIS-F | 0.3 | 0.06 | 0.10 | 0.4 | 0.09 | 0.10 |
| Δ SD - Improvements between HA and M12 FU in units of standard deviations; Ind. Weight - weight of individual indicator; Dim. Weight - Final weight of the sub-dimension of each indicator **^1^**Ind. weights are derived by dividing the individual standard deviations by the sum of all standard deviations. **^2^**Then the mean of the individual weights is calculated for a sub-dimension of the PRO-CM and rounded to one decimal place | | | | | | |

References

1. Herdman M, Gudex C, Lloyd A, Janssen M, Kind P, Parkin D, et al. Development and preliminary testing of the new five-level version of EQ-5D (EQ-5D-5L). Qual Life Res. 2011;20:1727–36. doi:10.1007/s11136-011-9903-x.

2. EQ-5D instruments. EQ-5D-5L – EQ-5D. https://euroqol.org/eq-5d-instruments/eq-5d-5l-about/. Accessed 15 Jul 2021.

3. van Hout B, Janssen MF, Feng Y-S, Kohlmann T, Busschbach J, Golicki D, et al. Interim scoring for the EQ-5D-5L: mapping the EQ-5D-5L to EQ-5D-3L value sets. Value Health. 2012;15:708–15. doi:10.1016/j.jval.2012.02.008.

4. Oppe M, Devlin NJ. EQ-5D value sets: inventory, comparative review and user guide: Springer; 2007.

5. Roos EM, Lohmander LS. The Knee injury and Osteoarthritis Outcome Score (KOOS): from joint injury to osteoarthritis. Health Qual Life Outcomes. 2003;1:64. doi:10.1186/1477-7525-1-64.

6. PROMIS: Patient-Reported Outcomes Measurement Information System - Home Page. 2013. https://commonfund.nih.gov/promis/index. Accessed 13 Oct 2021.

7. National Institutes of Health. PROMIS domain framework/definitions. 2018. https://www.healthmeasures.net/explore-measurement-systems/promis/intro-to-promis. Accessed 16 Jun 2021.

8. Singh JA, Lewallen DG. Depression in primary TKA and higher medical comorbidities in revision TKA are associated with suboptimal subjective improvement in knee function. BMC Musculoskelet Disord. 2014;15:127. doi:10.1186/1471-2474-15-127.

9. ICHOM. hip & knee osteoarthritis Data Collection reference guide. 2017.

10. Tiftikçioğlu Bİ. Multiple Sclerosis Functional Composite (MSFC): Scoring Instructions. Noro Psikiyatr Ars. 2018;55:S46-S48. doi:10.29399/npa.23330.

11. Gan X, Fernandez IC, Guo J, Wilson M, Zhao Y, Zhou B, Wu J. When to use what: Methods for weighting and aggregating sustainability indicators. Ecological Indicators. 2017;81:491–502. doi:10.1016/j.ecolind.2017.05.068.

12. Barclay M, Dixon-Woods M, Lyratzopoulos G. The problem with composite indicators. BMJ Qual Saf. 2019;28:338–44. doi:10.1136/bmjqs-2018-007798.

13. Talukder B, W. Hipel K, W. vanLoon G. Developing Composite Indicators for Agricultural Sustainability Assessment: Effect of Normalization and Aggregation Techniques. Resources. 2017;6:66. doi:10.3390/resources6040066.

14. Shwartz M, Restuccia JD, Rosen AK. Composite Measures of Health Care Provider Performance: A Description of Approaches. Milbank Q. 2015;93:788–825. doi:10.1111/1468-0009.12165.

15. Babbie ER. The practice of social research. Boston, MA: Cengage; 2021.

16. Nardo M, Saisana M, Saltelli A, Tarantola S. Tools for Composite Indicators Building: Ispra; 2005.

17. Ayton D, Soh S-E, Morello R, Ahern S, Earnest A, Brennan A, et al. Development of a percutaneous coronary intervention patient level composite measure for a clinical quality registry. BMC Health Services Research. 2020;20:44. doi:10.1186/s12913-019-4814-6.

18. Beierlein V. Ergebnismessung in der Orthopädie - Ergebnisberichte.

19. OECD. Handbook on constructing composite indicators: Methodology and user guide. Paris: OECD; 2008.

20. Hays RD, Bjorner JB, Revicki DA, Spritzer KL, Cella D. Development of physical and mental health summary scores from the patient-reported outcomes measurement information system (PROMIS) global items. Qual Life Res. 2009;18:873–80. doi:10.1007/s11136-009-9496-9.

21. Rosato R, Testa S, Bertolotto A, Confalonieri P, Patti F, Lugaresi A, et al. Development of a Short Version of MSQOL-54 Using Factor Analysis and Item Response Theory. PLoS One. 2016;11:e0153466. doi:10.1371/journal.pone.0153466.

22. Vickrey BG, Hays RD, Harooni R, Myers LW, Ellison GW. A health-related quality of life measure for multiple sclerosis. Qual Life Res. 1995;4:187–206. doi:10.1007/BF02260859.

23. Zaslavsky AM, Shaul JA, Zaborski LB, Cioffi MJ, Cleary PD. Combining health plan performance indicators into simpler composite measures. Health Care Financ Rev. 2002;23:101–15.

24. Tucker LR MRC. Exploratory factor analysis; 1997.

25. Metge C, Chateau D, Prior H, Soodeen R, De Coster C, Barré L. Composite measures/indices of health and health system performance; 2009.

26. MacCallum RC, Widaman KF, Zhang S, Hong S. Sample size in factor analysis. Psychological Methods. 1999;4:84–99. doi:10.1037/1082-989X.4.1.84.
